# Measurement of horizontal ocular deviation on magnetic resonance imaging in various disease with acute vertigo

**DOI:** 10.1371/journal.pone.0224605

**Published:** 2019-10-31

**Authors:** Yeon-Jun Yang, Ji Eun Choi, Min Tae Kim, Sang Hyub Kim, Min Young Lee, Dong Soo Yoo, Jae Yun Jung

**Affiliations:** 1 Department of Otorhinolaryngology-Head & Neck Surgery, Dankook University Hospital, Cheon-an, Republic of Korea; 2 Department of Otorhinolaryngology-Head & Neck Surgery, College of Medicine, Dankook University, Cheon-an, Republic of Korea; 3 Department of Radiology, College of Medicine, Dankook University, Cheon-an, Republic of Korea; University College London, UNITED KINGDOM

## Abstract

In our previous study, we found that horizontal ocular deviation (OD) was significantly increased in patients with unilateral vestibular neuritis (VN). This study is aimed to compare the measurements of horizontal OD in various diseases which can present as acute vertigo in the emergency department. We retrospectively reviewed patients who visited the emergency department and underwent brain MRI due to acute vertigo. We compared them to healthy controls who underwent brain MRI for a regular health examination. Among the study participants, 149 patients who were diagnosed with benign paroxysmal positional vertigo (BPPV), unilateral Ménière's disease (MD), vestibular migraine (VM), unilateral vestibular neuritis (VN), or posterior inferior cerebellar artery (PICA) infarction were enrolled. Absolute angles of horizontal OD were larger in the definite MD (19.1 ± 12.7°), possible and probable MD (15.5 ± 11.7°), and VN (22.2 ± 11.7°) groups compared to the control group (4.3 ± 3.7°). Most VN patients (83.3%) had horizontal OD toward the direction of the lesion. About half of the MD patients (46.2%) and half of the patients with PICA infarction (50.0%) had horizontal OD toward the opposite direction of the lesion. Regarding PICA infarction, horizontal OD was observed only in patients who immediately underwent an MRI after developing the PICA territory vestibulocerebellar infarction. Although the exact mechanism of horizontal OD is unclear, this study suggests that horizontal OD reflects a static vestibular imbalance, and that the eyeball is deviated to the weaker of the two vestibular nuclei during neural resting activity. Therefore, horizontal OD could be helpful in assessing for a prior vestibular imbalance.

## Introduction

Acute vertigo accounts for 12% of neurological presentations to the emergency department [1]. Most cases are caused by benign diseases of the inner ear, but about 10% of patients with cerebellar stroke may initially present with acute vertigo that mimics vestibular neuritis (VN) [2–6]. Recently, brain MRI has been increasingly used to diagnose small strokes in emergency departments, especially in clinics without trained otologists or neurologists [2,7]. In a previous study, we found that the horizontal angle of ocular deviation (OD) on a T2-weighted image was significantly increased in patients with unilateral vestibular neuritis (VN), compared to healthy controls [8]. In VN patients, the direction of the OD was toward the affected ear. The absolute angle of the horizontal OD significantly correlated with the severity of the vestibular imbalance, based on vestibular function tests. The results of this previous study suggested that the horizontal OD of a brain MRI could provide useful information for assessing vestibular imbalance at the time of acute vertigo. This study aimed to compare the measurements of horizontal OD in various diseases which can present as acute vertigo in the emergency department.

## Methods

### Ethics statement

The retrospective study protocol was approved by the Dankook University Hospital Institutional Review Board, and it was performed in accordance with relevant guidelines and regulations. All data were fully anonymized before analyzing and the Institutional Review Board waived the requirement for informed consent.

### Subjects and study design

From January 2015 to June 2016, medical records of all patients who visited the emergency department of the Dankook University Hospital due to acute vertigo were reviewed. Among them, patients who underwent a brain MRI in the emergency department were included. We excluded patients who had other neurologic symptoms, such as focal weakness, sensory change, ataxia, or dysarthria. To compare the ODs of various diseases, we included patients who were diagnosed with benign paroxysmal positional vertigo (BPPV), unilateral Ménière's disease (MD), vestibular migraine (VM), or unilateral vestibular neuritis (VN) [9–12]. Patients were diagnosed by an otologist within one week of undergoing MRI. Healthy controls (individuals who underwent a brain MRI for a regular health check-up in the same period) were randomly enrolled. Additionally, we included patients who visited for acute vertigo and were diagnosed with posterior inferior cerebellar artery (PICA) infarction by a neurologist between January 2011 and December 2016. All enrolled patents with PICA infarction initially had no other neurologic symptoms except for headache and unsteadiness.

### MRI protocols

All MRI examinations were conducted using a 1.5T scanner (Signa HDxt, GE Medical Systems, Milwaukee, WI, USA) with an 8-channel head coil. Before obtaining the MR images, each subject was instructed not to move their head, but to open or close their eyes freely. Because each MRI was performed in a dark room, it is likely that there were few visual fixation effects, regardless of whether a patient’s eyes were opened or closed. Among the routine MR imaging protocols, axial planes of T2-weighted fast-spin echo (FSE) images were selected to measure the horizontal OD. T2-weighted FSE images were obtained using the following parameters: TR/TE of 4550/103 ms; 23 sections; section thickness of 5 mm; intersection gap of 1.5 mm; matrix of 320 × 256; ETL of 23; FOV of 240 × 240 mm; NEX of 1; and an acquisition time of about two minutes.

### Definition of horizontal ocular deviation (OD)

**Fig 1:**
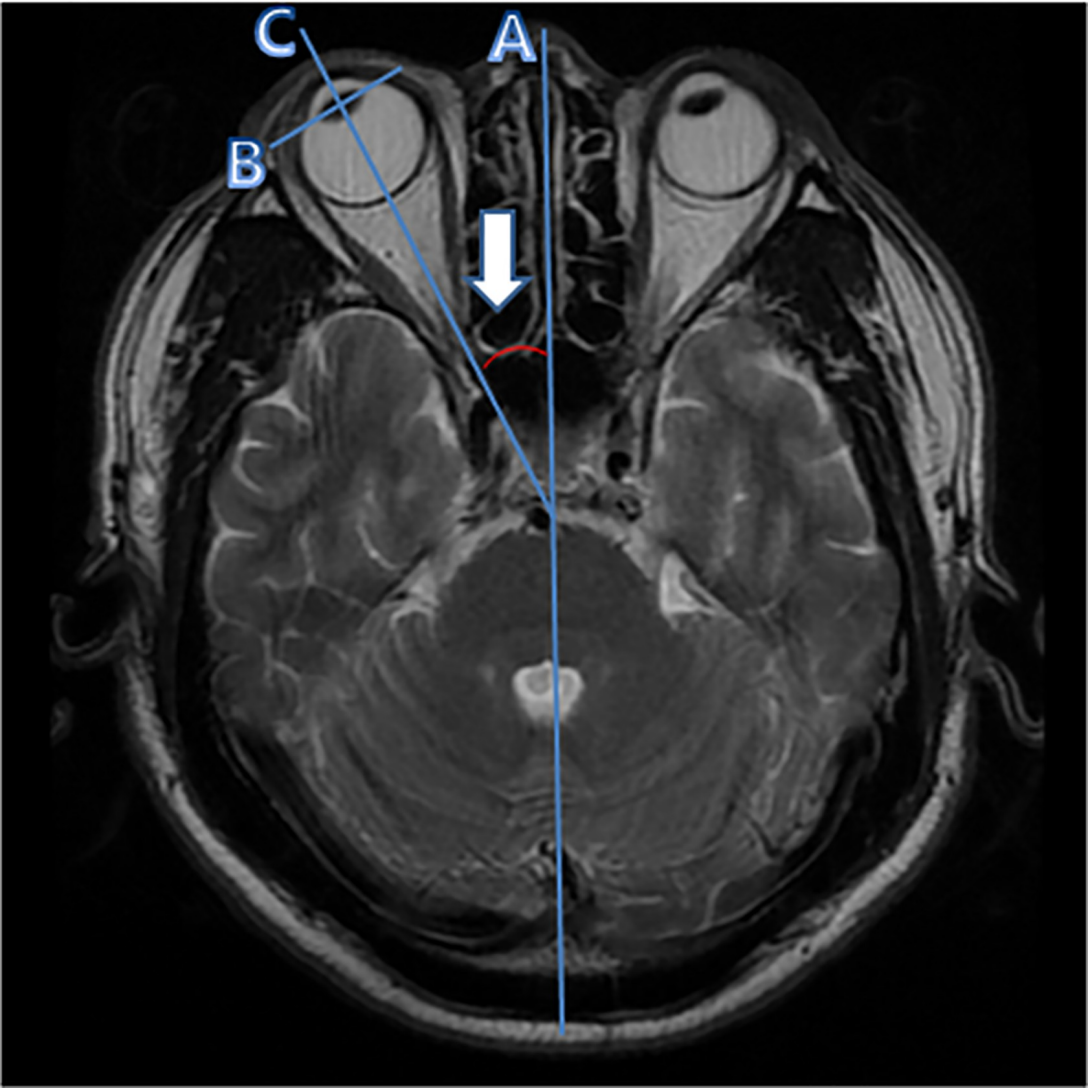
Ocular deviation (OD) measurements in T2-weighted axial images of brain MRIs. Three lines were drawn to measure OD values in T2-weighted axial images. The first line (A) was drawn on the midline of the nasal structures. The second line (B) connected both ends of the lens. The third line (C) was drawn perpendicular to line B. We measured the angle formed by the intersection of lines A and C (arrow).

Horizontal OD was calculated by a method previously reported (Fig 1) [13]. The first line (A) was drawn anteroposteriorly through the midline nasal structures; line B was drawn through both ends of the lenses (separately); and line C was drawn perpendicularly to line B. The OD angle was calculated for the right and left eyes by measuring the angle formed by the intersection of lines A and C. The OD angle was recorded as a positive number if the eye was deviated to the right side, and a negative number if the eye was deviated to the left side. The OD angle in each subject was defined as an average of right and left OD angles.

### Direction of horizontal ocular deviation (OD)

After determining optimal diagnostic cutoff value for significant horizontal OD, the direction of the horizontal OD divided into three categories; no deviation, same direction, and opposite direction. If the absolute horizontal OD angle was smaller than cutoff value, we defined it as a no deviation. If the absolute horizontal OD angle was larger than cutoff value and its direction of OD was toward the lesion side, we defined it as the ‘same direction.’ Similarly, if the absolute horizontal OD angle was larger than cutoff value and its direction of OD was away from the lesion side, we defined it as the ‘opposite direction.’

### Statistical analyses

All data were analyzed using SPSS 20.0 (SPSS Inc., Chicago, IL, USA). To compare the absolute values of horizontal OD between patients with acute vertigo (six different groups) and healthy controls, a one-way analysis of variance (ANOVA) or Kruskal-Wallis test was conducted (depending on the outcome of a normality assumption test). If there was a significant difference among two groups, a post-hoc independent t-test or Mann-Whitney test was performed to evaluate the differences between them. We used an adjusted *p*-value of 0.008 (*i*.*e*. 0.05/6), based on a Bonferroni correction. Receiver operating characteristic (ROC) curve analyses were performed to estimate the optimal absolute angle of OD for assessing acute unilateral vestibulopathy. Each ROC curve was plotted using measures of sensitivity and specificity based on various anthropometric cutoff values. The direction of OD in acute unilateral vestibulopathy was compared with that of BPPV, MD, and PICA infarction using Pearson's chi-squared test or Fisher's exact test. The adjusted *p*-value was 0.0166 (*i*.*e*. 0.05/3), based on a Bonferroni correction.

## Results

### Demographics of the study population

A total of 149 patients with acute vertigo and 30 healthy controls were retrospectively enrolled. Demographic characteristics are shown in Table 1. The study population was classified according to diagnosis as follows: 1) healthy controls (*n* = 30), 2) benign paroxysmal positional vertigo (BPPV, *n* = 51), 3) definite Ménière's disease (dMD, *n* = 16), 4) possible or probable Ménière's disease (pMD, *n* = 10), 5) vestibular migraine (VM, *n* = 28), 6) vestibular neuritis (VN, *n* = 30), and 7) PICA infarction (*n* = 14).

**Table 1:** Demographic data of the study population.

Diagnosis	Number	Age (years)	Sex (M : F)
Control	30	43.8 ± 9.8	20 : 10
BPPV	51	55.6 ± 14.5	25 : 26
Definite MD (dMD)	16	54.8 ± 13.2	9 : 7
Probable or Possible MD (pMD)	10	54.6 ± 7.0	1 : 9
Vestibular Migraine (VM)	28	48.6 ± 12.7	8 : 20
Vestibular Neuritis (VN)	30	54.9 ± 12.3	18 : 12
PICA Infarction	14	63.4 ± 9.4	13 : 1

### The absolute angles of horizontal OD

**Fig 2:**
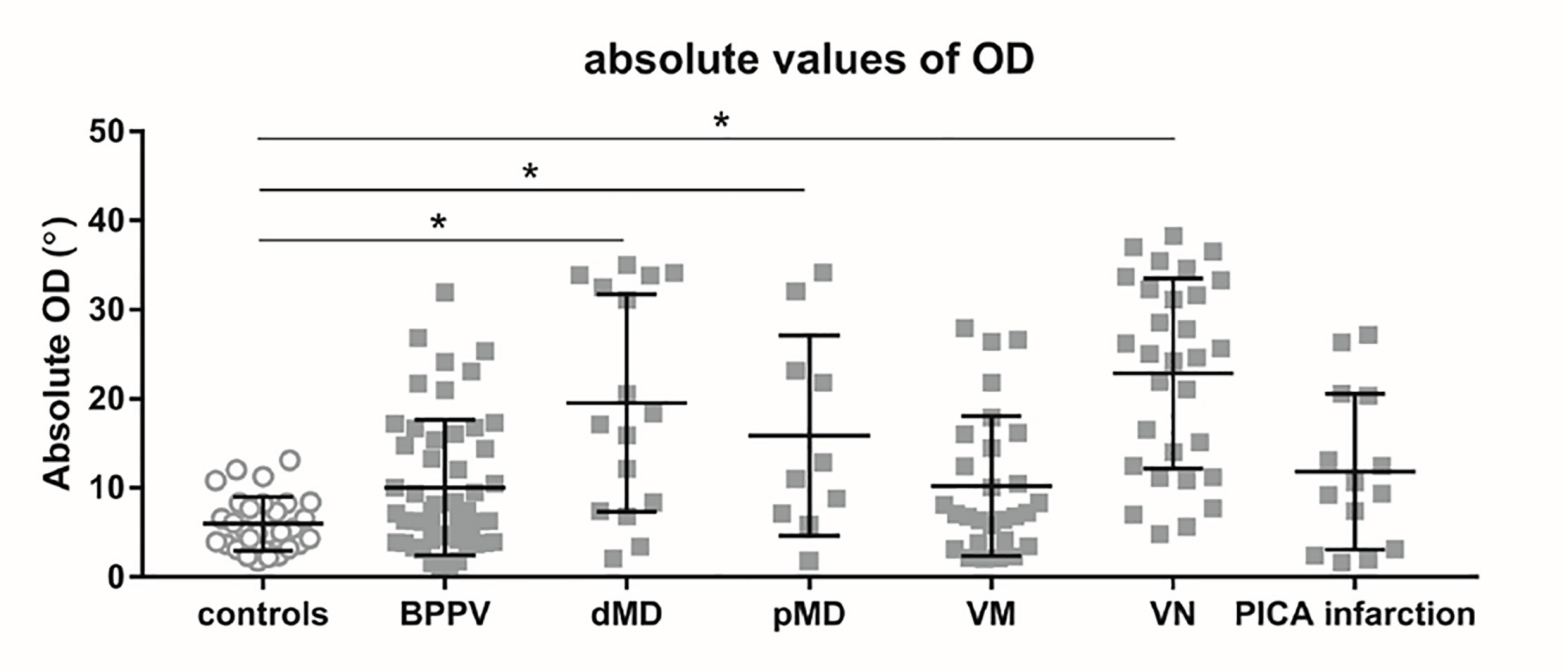
The absolute values of horizontal ocular deviation (OD) for various diseases. Results of scatter plots for healthy controls and patients with acute vertigo (caused by various diseases) are shown as circles (●) and squares (■), respectively. Bars and error bars represent mean absolute OD and standard deviation, respectively. An asterisk (*) indicates a significant difference between two groups (*i*.*e*. controls and patients with acute vertigo), based on a post-hoc analysis. The adjusted *p*-value was 0.05/6, based on a Bonferroni correction.

**Fig 3:**
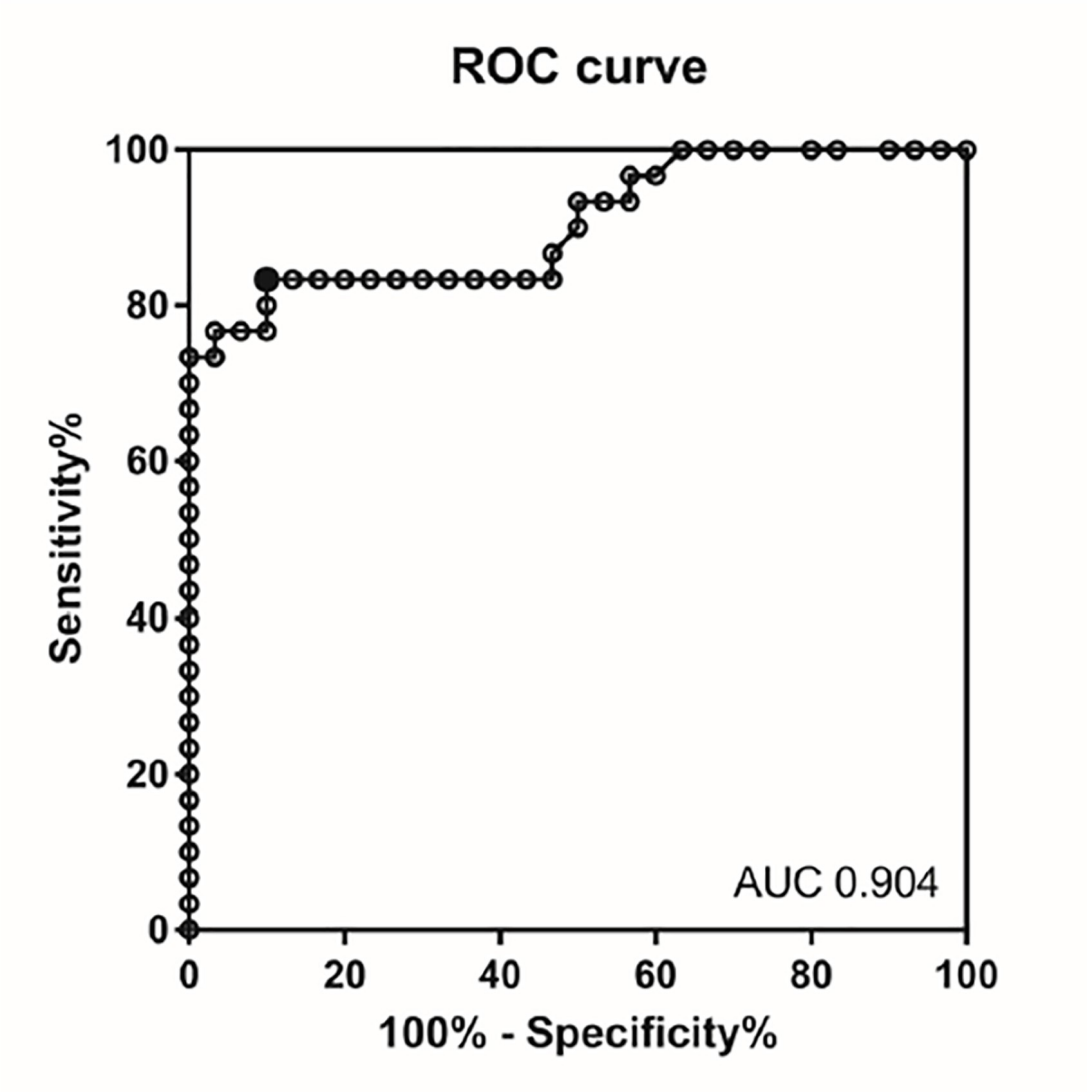
Receiver operating characteristic (ROC) curve analyses for estimating the optimal absolute angle of ocular deviation (OD). The area under the ROC curve (ACU) was 0.904. A cutoff of ≥ 9.625° had a sensitivity of 83.3% and a specificity of 90.0%.

The absolute angles of horizontal OD for the various diseases are shown in Fig 2. The absolute horizontal OD angles were 4.3 ± 3.7° (control), 8.6 ± 8.3° (BPPV), 19.1 ± 12.7° (dMD), 15.5 ± 11.7° (pMD), 8.9 ± 8.5° (VM), 22.2 ± 11.7° (VN), and 11.8 ± 8.7° (PICA infarction). Compared to the control group, the absolute angles of horizontal OD were significantly larger in the dMD, pMD, and VN groups. However, the absolute angles of horizontal OD were similar to the control group in the BPPV, VM, and PICA infarction groups. The ROC curve for the control and VN absolute angles of horizontal OD is shown in Fig 3. We plotted sensitivity (%) as a function of specificity (100%—specificity). A cutoff of ≥ 9.625° yielded a sensitivity of 83.3% and a specificity of 90%. That is, a horizontal OD of more than 9.625° meant that a subject tended to have a static vestibular tone imbalance (area under the curve [AUC] = 90.4%, *p* <0.001).

### Direction of horizontal OD

Table 2 shows the direction of horizontal OD for VN, BPPV, MD, and PICA infarction. If the absolute horizontal OD angle was smaller than 9.625°, we defined it as a no deviation. Of 30 patients with VN, 5 patients (13.6%) showed no deviation and 25 patients (83.3%) showed significant deviation. Among VN patient with significant deviation (*n* = 25), all of patients had significant deviation toward the same direction as the lesion side. No patient with VN had significant deviation toward the opposite direction. Compared to the VN group, the BPPV, MD, and PICA infarction groups showed a significantly different direction of horizontal OD (all p-values < 0.001). Of 51 patients with BPPV, 34 patients (66.7%) had no deviation. Only 17 patients with BPPV showed significant deviation either in the same direction (*n* = 10, 19.6%) or in the opposite direction (*n* = 7, 13.6%). Similar to VN patients, of 26 patients with MD, most patients (*n* = 17, 65.4%) had significant deviation and 9 patients (34.6%) had no deviation (34.6%). However, among 17 patients who had significant deviation, 12 patients with MD (46.2%) had horizontal OD to the opposite direction and 5 patients with MD (19.2%) had horizontal OD to the same direction. Of 14 patients with PICA infarction, half of the patients (50%) showed no OD, whereas the other half showed horizontal OD toward the opposite side of the lesion.

**Table 2:** Direction of the horizontal ocular deviation (OD).

Diagnosis	No deviation	Significant deviation		Adjusted *p*-value
		Same direction	Opposite direction	
VN	5 (16.7%)	25 (83.3%)	0 (0%)	ref
BPPV	34 (66.7%)	10 (19.6%)	7 (13.7%)	< 0.001
MD	9 (34.6%)	5 (19.2%)	12 (46.2%)	< 0.001
PICA Infarction	7 (50%)	0 (0%)	7 (50%)	< 0.001

Pearson's chi-squared test or Fisher’s exact test were used to compare the direction of OD in various diseases (BPPV, MD, and PICA infarction) with that in VN. The adjusted *p*-value was 0.0166 (*i*.*e*. 0.05/3), based on a Bonferroni correction.

### The horizontal OD of PICA infarction

Table 3 shows general information for the PICA infarction patients. Most patients with PICA infarction had spontaneous nystagmus, and the direction of nystagmus was toward the lesion side. All of the PICA infarction territory involved the vestibulocerebellum (*i*.*e*. the flocculus, tonsils, nodulus, and uvula), except for one patient (PICA11). In PICA infarction, a horizontal OD tended to be found in patients who immediately underwent an MRI after developing dizziness.

**Table 3:** Demographic data of the study population.

Patients	Absolute angle of OD (°)	OD	Nystagmus	Initial symptoms	MRI findings						
					Interval time	Lesion side	Involved structures (cerebellum)				
							flocculus	nodulus	uvula	tonsil	posterior lobe
PICA1	9.35	No deviation	LB	Dz, HA, US	5 days	Left	+	+		+	+, large
PICA2	9.2	No deviation	ND	Dz	1 day	Left		+		+	+, large
PICA3	1.95	No deviation	RB	Dz, HA	7 days	Right		+		+	
PICA4	1.65	No deviation	LB	Dz, US	1 day	Left				+	+, multifocal
PICA5	3.1	No deviation	RB	Dz	4 days	Right		+		+	+, large
PICA6	7.4	No deviation	DB, GEN	Dz, HA, US	4 days	Left			+	+	+, small
PICA7	2.45	No deviation	No nystagmus	US	5 days	Left				+	+, large
PICA8	12.45	OD to opposite side	RB	Dz	0 days	Right		+	+		
PICA9	13.1	OD to opposite side	No nystagmus	HA, Dz, US	0 days	Left		+	+	+	+, small
PICA10	27.2	OD to opposite side	RB	Dz, US	2 days	Right		+		+	+, large
PICA11	20.55	OD to opposite side	LB	US	0 days	Left					+, small
PICA12	20.3	OD to opposite side	LB	Dz, US	0 days	Left		+	+		+, small
PICA13	26.3	OD to opposite side	ND	Dz, US	2 days	Right				+	+, small
PICA14	10.6	OD to opposite side	RB	Dz, US	0 days	Right		+	+	+	+, large

The interval time defines the time it took to perform an MRI after the patient experienced dizziness. OD, ocular deviation; Dz, dizziness; US, unsteadiness; HA, headache; ND, not done; RB, right beating nystagmus; LB, left beating nystagmus; DB, down beating nystagmus; GEN, gaze evoked nystagmus.

## Discussion

This study measured horizontal OD in patients with BPPV, MD, VM, VN, and PICA infarction. A previous study has demonstrated that the absolute horizontal OD angle in patients with VN was significantly greater than that observed in controls [14]. The absolute angle of horizontal OD in VN patients significantly correlated with the following: slow phase velocity (spontaneous nystagmus); canal paresis (caloric test); gain and phase (rotatory chair test); and interaural amplitude difference (vestibular evoked myogenic potential, or VEMP). Although the mechanism of OD observed with MRI is not fully understood, we presumed that horizontal OD is related to bias of the perceived horizontal position due to vestibular tone imbalance. When a clinician encounters a patient, who has experienced vestibular recovery or compensation after an acute vertigo, a horizontal OD could be helpful in assessing for a previous vestibular imbalance. In this study, compared to controls, the absolute angle of horizontal OD was also significantly larger in patients with MD, which can cause a vestibular imbalance (Fig 2). However, these were similar to the controls in patients with BPPV or VM, which do not cause a vestibular imbalance. These results support the notion that a horizontal OD observed by MRI reflects a vestibular imbalance at the time of acute vertigo.

In this study, the direction of horizontal OD was toward the weaker of the two vestibular nuclei during neural resting activity (Table 2). In the acute stage of VN, an asymmetry of neural activity is caused by a loss of vestibular resting discharge. Thus, the direction of horizontal OD is toward the lesion side in VN. While, in the acute stage of MD, it is presumed that rupture of the membranous labyrinth initially excites the vestibular afferent nerve fibers, producing an asymmetry of neural activity [15]. If the MD patients are in the acute stage, their direction of the horizontal OD will be opposite the lesion. In addition to peripheral type of vestibular imbalance, half of patients with PICA infarction also showed significant horizontal OD in the opposite direction of the lesion. Previous studies have reported that patients with PICA infarction in the medial branch (mPICA) show spontaneous nystagmus mimicking acute peripheral vestibulopathy [16,17]. The medial branch supplies the nodulus and uvula, which are key components of the vestibulocerebellum [18,19]. The vestibulocerebellum has strong connections with the vestibular nucleus, and Purkinje fibers from the vestibulocerebellum have an inhibitory effect on the ipsilateral vestibular nucleus. Thus, the horizontal OD could be directed away from the PICA infarction by an increased tonic activity of ipsilesional vestibular nucleus neurons (due to a disconnection of inhibitory Purkinje fibers from the vestibular nucleus). In the current study, almost PICA infarction territory involved the vestibulocerebellum (Table 3). Only one patient (PICA11) had focal infarction in the left posterior lobe of the cerebellar hemisphere; this was due to embolism by cardiac myxoma. This patient also showed right canal paresis on caloric testing, which indicated right vestibulopathy. Thus, nystagmus and horizontal OD observed in this patient are thought to be caused by vestibulopathy rather than PICA infarction. Significant horizontal OD was observed in patients who immediately underwent an MRI after developing a PICA territory vestibulocerebellar infarction. Thus, our findings suggest that horizontal OD may be caused by an acute stage of vestibular neural asymmetry in vestibulocerebellum.

The mechanism of horizontal OD observed with MRI in patients with vestibular imbalance is not fully understood. If the nystagmus observed as a horizontal OD in brain MRI, these periodic eyeball movement would produce motion artifact such as blurring. However, compared to images of patients with congenital nystagmus (S1 Fig), relatively clear images of the eyeball could be obtained in this study. Therefore, we presumed that a clear horizontal OD image might be caused by a bias in the position of the head, causing a vestibular tone imbalance between the two vestibular nuclei during neural resting activity. Previous studies have demonstrated that the subjective visual horizontal position in darkness is ipsilesionally biased in subjects with unilateral vestibulopathy [20–22]. Because each MRI was performed in a dark room, there was little visual feedback, regardless of whether a patient’s eyes were opened or closed. In the absence of visual feedback, it is possible that a bias in the perceived horizontal position due to a vestibular imbalance may have resulted in a horizontal OD.

In conclusion, this study has shown that horizontal OD reflects the vestibular imbalance in various diseases, and that the eyeball deviates to the weaker of the two vestibular nuclei during neural resting activity. Although horizontal OD is unlikely to help with a differential diagnosis, using T2-weighted MRI could provide helpful information in assessing for a prior vestibular imbalance in patients with restored or compensated static vestibular imbalance.

## Supporting information

S1 Fig Examples of ocular deviation (OD) in congenital nystagmus patients. A motion artifact of eyeball, due to nystagmus, was observed using T2-weighted MRI.
(TIF)
