# Supplementary figures and images for: Measurement of horizontal ocular deviation on magnetic resonance imaging in various disease with acute vertigo
Source: PLoS One. 2019 Oct 31;14(10):e0224605. doi: 10.1371/journal.pone.0224605 (PMC6822736; doi:10.1371/journal.pone.0224605)

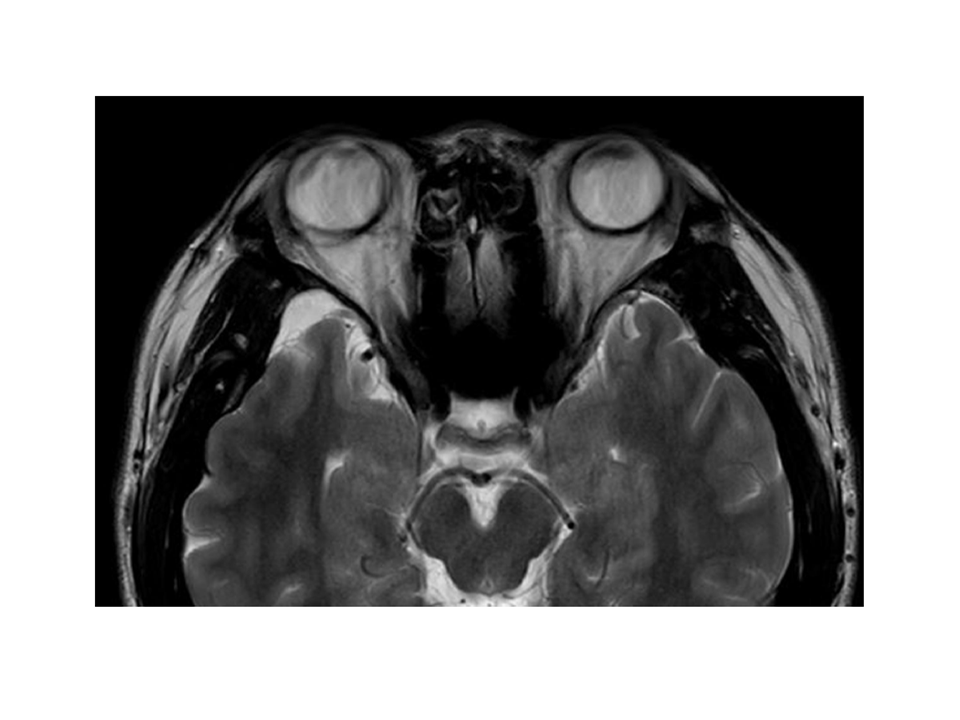

Supplement: S1 Fig — A motion artifact of eyeball, due to nystagmus, was observed using T2-weighted MRI. (TIF) [file pone.0224605.s001.TIF]
